# Combined robotic approach and enhanced recovery after surgery pathway for optimization of costs in patients undergoing proctectomy

**DOI:** 10.1002/bjs5.50281

**Published:** 2020-04-30

**Authors:** P. Rouanet, A. Mermoud, M. Jarlier, N. Bouazza, A. Laine, H. Mathieu Daudé

**Affiliations:** ^1^ Surgical Oncological Department Montpellier France; ^2^ Biometrics Unit Montpellier France; ^3^ Financial Department Montpellier France; ^4^ Clinical Research and Innovation Department Montpellier France; ^5^ Health Information Department, Montpellier Cancer Institute University of Montpellier Montpellier France

## Abstract

**Background:**

Enhanced recovery after surgery (ERAS) pathways are beneficial in proctocolectomy, but their impact on robotic low rectal proctectomy is not fully investigated. This study assessed the impact of an ERAS pathway on the outcomes and cost of robotic (RTME) *versus* laparoscopic (LTME) total mesorectal excision.

**Methods:**

A retrospective review was performed of patients with rectal cancer in a single French tertiary centre for three yearly periods: 2011, LTME; 2015, RTME; and 2018, RTME with ERAS. Patient characteristics, operative and postoperative data, and costs were compared among the groups.

**Results:**

A total of 220 consecutive proctectomies were analysed (71 LTME, 58 RTME and 91 RTME with ERAS). A prevalence of lower and locally advanced tumours was observed with RTME. The median duration of surgery increased with the introduction of RTME, but became shorter than that for LTME with greater robotic experience (226, 233 and 180 min for 2011, 2015 and 2018 respectively; *P* < 0·001). The median duration of hospital stay decreased significantly for RTME with ERAS (11, 10 and 8 days respectively; *P* = 0·011), as did the overall morbidity rate (39, 38 and 16 per cent; *P* = 0·002). Pathology results, conversion and defunctioning stoma rates remained stable. RTME alone increased the total cost by €2348 compared with LTME. The introduction of ERAS and improved robotic experience decreased costs by €1960, compared with RTME performed in 2015 without ERAS implementation. In patients with no co‐morbidity, costs decreased by €596 for RTME with ERAS *versus* LTME alone.

**Conclusion:**

ERAS is associated with cost reductions in patients undergoing robotic proctectomy.

## Graphical abstract

Managing robotic total mesorectal excision (RTME) using an enhanced recovery after surgery (ERAS) programme in patients with rectal cancer decreased overall costs compared with RTME alone. The combination significantly decreased operating time, duration of hospital stay and global morbidity compared with RTME and laparoscopic total mesorectal excision alone.

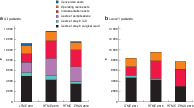

ERAS and robotic proctectomy for rectal cancer

## Abstract (es)

**Antecedentes:**

Las vías clínicas ERAS son beneficiosas en la proctocolectomía, pero su impacto en la proctectomía rectal baja robótica no se ha investigado exhaustivamente. El objetivo de este estudio fue evaluar el impacto de la vía clínica ERAS sobre los resultados y el coste de la proctectomía robótica (resección total del mesorrecto robótica, *robotic total mesorectal excision*, RTME) *versus* procedimientos laparoscópicos de resección total del mesorrecto (*laparoscopic total mesorectal excision*, LTME).

**Métodos:**

Revisión retrospectiva de pacientes con cáncer de recto tratados en un único centro terciario francés durante un periodo de tres años: 1) 2011: resección total del mesorrecto laparoscópica (LTME); 2) 2015: TME robótica y 3) 2018: TME robótica plus ERAS. Se compararon las características de los pacientes, los datos operatorios y postoperatorios, y los costes entre subgrupos utilizando análisis estadísticos.

**Resultados:**

Se analizaron 220 proctectomías consecutivas que incluían 71 LTME, 58 RTME y 91 RTME plus ERAS. Se observó un predominio de tumores inferiores y localmente avanzados en la RTME. La mediana del tiempo operatorio aumentó con la introducción de RTME, pero llegó a ser inferior que en la LTME con una mayor experiencia robótica (226, 233 y 180 minutos para los periodos 1, 2 y 3, respectivamente; *P* = 0,0001). La mediana de la estancia hospitalaria disminuyó significativamente con la RTME plus ERAS (11, 10 y 8 días; *P* = 0,01), así como la morbilidad global (40%, 38% y 16%; *P* = 0,002). Los resultados de la anatomía patológica, las tasas de conversión y de estomas de protección permanecieron estables. La RTME sola aumentó el coste total en €2.348 comparado con la LTME. La introducción de ERAS y una mejora en la experiencia robótica disminuyeron los costes en €1.960 *versus* RTME realizada en 2015 sin la implementación de ERAS. En pacientes sin comorbilidades, los costes disminuyeron en €1.196 con RTME plus ERAS *versus* LTME sola.

**Conclusión:**

ERAS se asocia con reducciones de coste en la proctectomía robótica.

## Introduction

Robotic total mesorectal excision (RTME) in patients with rectal cancer can provide several advantages compared with laparoscopic total mesorectal excision (LTME), including the use of a stable three‐dimensional camera, wristed instrumentation, and ease of dissection in narrow spaces such as in male and fatty pelvis^1^. Nevertheless, evidence is sparse and robust prospective studies are needed to demonstrate the benefits of RTME^2^. A potential disadvantage of robotic surgery is its associated costs. The ROLARR phase III trial^3^ estimated that costs were around €1020 higher for RTME than for LTME (*P* = 0·02); the main drivers of this difference were a longer mean duration of surgery and the mean cost of robotic instruments^3^. Literature reviews have underlined the difficulties involved in conducting robust medicoeconomic studies owing to the heterogeneity of patients and differences in operative and postoperative management^4^, ^5^, ^6^.

This paper aimed to compare patients with rectal cancer undergoing a sphincter‐saving procedure using a standard LTME or RTME technique with or without an enhanced recovery after surgery (ERAS) pathway. The primary objective was to evaluate the impact of ERAS on the outcomes and costs of robotic proctectomy.

## Methods

This study was conducted at the Montpellier Cancer Institute, where robotic rectal surgery was introduced in 2012, followed by ERAS management in 2016 to improve efficiency and patient benefits. After obtaining approval from the institutional review board, a retrospective evaluation was conducted of patients with rectal cancer who had resection for rectal carcinoma. Inclusion criteria were sphincter‐saving surgery with or without a defunctioning stoma. Data were taken from the Programme de Médicalisation des Systèmes d'Information (PMSI), a prospective database that provides information for all French hospitals on the volume of operations per surgical approach with the corresponding length of hospital stay (LOS).

Consecutive patients were categorized into three cohorts according to the year of procedure (2011, 2015 and 2018), and type of surgical and perioperative management. An interval between these years was considered to avoid the learning curves associated with the introduction of both the robotics and ERAS implementation.

Accordingly, the first group of patients underwent LTME and treatment during 2011, the second cohort had RTME (da Vinci® Si™ system; Intuitive Surgical Sàrl, Aubonne, Switzerland) during 2015, and the third group underwent RTME (da Vinci® XI; Intuitive Surgical Sàrl) plus an ERAS pathway during 2018.

The surgical procedures were standardized for all patients and done by the same team under a single senior surgeon, with no differences between groups. The RTME technique was as described previously^1^, ^7^, ^8^. ERAS management was completed according to the most recent guidelines^9^, ^10^, ^11^. According to institutional protocol, since 2010 a defunctioning stoma has not been performed systematically. Non‐stoma management was standardized when the resection was assessed as having no surgical difficulties, with good colonic preparation and a good quality of the anastomosis (perfect colonic vascularization, negative anastomotic test, no traction, complete doughnuts).

The following data were recorded and analysed in each group: patient characteristics (age, sex, BMI, tumour localization, T status, tumour stage, neoadjuvant treatment), duration of surgery, postoperative data (reoperation rate, conversion, pathological assessment of circumferential resection margin, LOS, rate of postoperative stoma), morbidity (fistula, stenosis, colonic necrosis, abscess and occlusion).

Patients in each cohort were further subdivided as level 1–2 (minor or no co‐morbidities) or level 3–4 (major co‐morbidities) according to ICD‐11, 2018 (https://www.atih.sante.fr/manuel-des-ghm-version-definitive-2018).

### Medical devices

The medical devices used for each surgical approach were compiled in a specific database and then valued according to purchase prices given by the hospital's pharmacy. Single‐use and sterilizable reusable medical devices were taken into account. For reusable devices, the cost was calculated by dividing the purchase price by the maximum number of uses recommended by the manufacturer. Sterilization costs for reusable medical devices were not included in the analysis, but these data are not significantly different between the two operative approaches.

### Determination of costs

The cost study was performed under the control of the French Department of Medical Information (DIM) and management controllers. In France, there is a lack of specific payment according to the technique (Groupe Homogène de Séjour – the billing information that defines the amount of money the hospital will receive to treat a specific patient). National LOS and instrument costs were derived from the National Cost Study (Etude Nationale des Coûts 2018^12^).

#### *Surgical costs*

The median duration of surgery was determined for each group. Duration of surgery involves room occupation, and includes the time before surgery when the patient receives anaesthesia, time required for the operation, and a short postsurgical time before transfer to the recovery room. Median duration of surgery, combined with the cost of the operating room per minute, gives the cost of the operating room for each surgical approach. The costs of robot‐assisted materials can be divided as follows: instrumentation, which includes reusable and disposable instruments (such as drapes, obturators and caps); capital costs (the cost of the robotic platform, which, as capital expenditure for the hospital, is depreciated on a 7‐year basis); and maintenance, including annual costs for service and maintenance of the robotic platform. Costs associated with depreciation and maintenance of the robotic platform were included in the cost per minute for the operating theatre, as they represent a significant proportion of this cost (7·8 per cent in 2015; 6·9 per cent in 2018).

#### *Conversion costs*

The conversion cost from minimally invasive to open surgery was estimated by multiplying the difference in LOS between the two surgical approaches (open and minimally invasive) by the cost of stay in the surgical ward and adding to the result the cost of the open surgery materials required in addition to the minimally invasive surgery resources. The national LOS for the different surgical approaches, as well as the additional instrumentation costs, were extracted from the French National Cost Studies^12^. To limit sample size bias, the conversion rates reported in a large 400‐patient study that had been conducted in the authors' hospital were used: 9·5 per cent for laparoscopy and 2 per cent for robot‐assisted surgical approaches^1^.

#### *Cost of hospital stay*

Mean LOS was taken from the DIM, and valued using the daily cost of a stay in the surgical ward at Montpellier Cancer Institute. ICU stays were also determined and valued. None of the patients analysed required the resuscitation unit.

#### *Complication costs*

The cost of complications per patient was estimated by multiplying the difference in LOS between level 3–4 patients (major co‐morbidities) and level 1–2 patients (minor/absence of co‐morbidities) by the cost of stay in the surgical ward increased by the extra consumable cost, and finally by the proportion of level 3–4 patients.

### *Statistical analysis*

Quantitative variables are reported as median (range) values. The non‐parametric Kruskal–Wallis test was used to compare sample distributions between the three groups (2011, 2015 and 2018). Pairwise cost comparisons were done using the non‐parametric Mann–Whitney two‐sample test. Qualitative variables are described by the number and frequency of observations for each of the outcomes. The χ^2^ test was used for comparison of the proportions. Analyses were carried out using Stata® software version 13.0 (StataCorp, College Station, Texas, USA).

## Results

Of 288 patients who had a proctectomy over the 3 years, 220 consecutive patients undergoing TME with a sphincter‐saving procedure were included in the study and analysed retrospectively. Sixty‐eight patients were excluded as their operation was not minimally invasive or not compliant with the ERAS protocol. Demographic data showed no differences in age, sex or BMI, but there were significant differences in tumour location and T category (*Table* 1). Middle‐third and low rectal tumors were more prevalent in the last period compared with the first period (83 *versus* 67 per cent respectively; *P* = 0·009), as were more advanced stages (T3–4: 90 *versus* 67 per cent respectively; *P* = 0·005). The rate of neoadjuvant treatment was comparable.

**Table 1:** Patient demographics

	LTME alone (2011) (*n* = 71)	RTME alone (2015) (*n* = 58)	RTME with ERAS (2018) (*n* = 91)	*P* †
**Age (years)** *	60 (35–85)	66 (30–86)	65 (32–91)	0·072‡
**Sex**				0·721
M	47 (66)	40 (69)	57 (63)	
F	24 (34)	18 (31)	34 (37)	
**BMI (kg/m** ^**2**^ **)** *	24·4 (16·8–35·8)	25·1 (16·9–40·1)	24·8 (17·4–36·0)	0·912‡
≤ 30	64 (90)	46 (79)	76 of 87 (87)	0·190
> 30	7 (10)	12 (21)	11 of 87 (13)	
**Rectal tumour location (cm)**	*n* = 69		*n* = 86	0·009
≥ 11 (high)	22 (32)	13 (22)	15 (17)	
6–10 (middle)	21 (30)	34 (59)	46 (53)	
≤ 5 (low)	26 (38)	11 (19)	25 (29)	
**T category (MRI)**	*n* = 58	*n* = 50	*n* = 81	0·005
T1	3 (5)	0 (0)	1 (1)	
T2	16 (28)	4 (8)	7 (9)	
T3	34 (59)	42 (84)	61 (75)	
T4	5 (9)	4 (8)	12 (15)	
**Neoadjuvant therapy**	45 (63)	43 (74)	65 (72)	0·340
Chemotherapy	8 (18)	5 (12)	13 (20)	
Chemoradiotherapy	36 (80)	30 (70)	39 (60)	
Both	1 (2)	8 (19)	13 (20)	

Values in parentheses are percentages unless indicated otherwise;

* values are median (range). LTME, laparoscopic total mesorectal excision; RTME, robotic total mesorectal excision; ERAS, enhanced recovery after surgery pathway.

† χ^2^ test, except

‡ Kruskal–Wallis test.

Implementation of the RTME–ERAS programme was 40 per cent in 2016, 54 per cent in 2017, and 86 per cent in 2018.

### Operative and postoperative data

The median duration of surgery increased by 7 min from LTME in 2011 to RTME in 2015, whereas RTME in 2018 showed a significant decrease of 46 min compared with LTME in 2011 (*P* < 0·001) (*Table* 2). There was no difference in pathology results between the three time periods in terms of reoperation rate (median 5 per cent) or positive circumferential resection margin less than 1 mm (median 10 per cent). The rate of 1‐month postoperative defunctioning stoma was also stable over time (median 53 per cent). LOS remained stable between LTME in 2011 and RTME in 2015 (10–11 days), but addition of ERAS management to RTME enabled a 2‐day reduction in LOS (*P* = 0·011) (*Tables* 2 and 3). The morbidity rate was significantly lower with RTME plus ERAS (16 per cent) compared with both LTME (39 per cent) and RTME (38 per cent) alone (*P* = 0·002) (*Table* 2).

**Table 2:** Operative results for proctectomy

	LTME alone (2011) (*n* = 71)	RTME alone (2015) (*n* = 58)	RTME with ERAS (2018) (*n* = 91)	*P* †
**Duration of surgery (min)** *	226 (115–428)	233 (140–374)	180 (118–395)	< 0·001‡, §
**Reoperation rate**	5 (7)	3 (5)	4 (4)	0·571
**CRM < 1 mm**	7 (10)	6 (10)	8 (9)	0·802
**Conversion**	6 (8)	3 (5)	3 (3)	0·356
**No defunctioning stoma**	35 (49)	24 (41)	43 (47)	0·495
**Length of stay (days)** *	11 (6–57)	10 (5–41)	8 (4–41)	0·011‡
**Morbidity**			*n* = 90	
None	43 (61)	36 (62)	76 (84)	0·002
Fistula	3 (4)	6 (10)	3 (3)	
Stenosis	0 (0)	2 (3)	0 (0)	
Necrosis	2 (3)	0 (0)	0 (0)	
Abscess	1 (1)	1 (2)	0 (0)	
Occlusion	11 (15)	4 (7)	0 (0)	
Other	11 (15)	9 (16)	11 (12)	

Values in parentheses are percentages unless indicated otherwise;

* values are median (range). LTME, laparoscopic total mesorectal excision; RTME, robotic total mesorectal excision; ERAS, enhanced recovery after surgery; CRM, circumferential resection margin.

† χ^2^ test, except

‡ Kruskal–Wallis test.

§ Pairwise comparisons: *P* = 0·839, LTME *versus* RTME; *P* < 0·001, LTME *versus* RTME + ERAS; *P* < 0·001, RTME *versus* RTME + ERAS (Mann–Whitney two‐sample test).

**Table 3:** Breakdown of costs associated with proctectomy

	LTME alone (2011)		RTME alone (2015)		RTME with ERAS (2018)	
	All (*n* = 71)	Level 1 (*n* = 26)	All (*n* = 58)	Level 1 (*n* = 35)	All (*n* = 91)	Level 1 (*n* = 25)
**Length of stay (days)** *						
Levels 1 and 2	10·02	9·38	8·50	8·49	7·39	6·44
Levels 3 and 4	17·25	n.a.	23·52	n.a.	17·04	n.a.
In intensive care ward	0·33	0·12	0·82	0·29	0·65	0·00
In continuous care ward	1·82	1·46	1·62	0·54	0·72	0·12
**Cost of stay (€/day)**						
In surgical ward	494	494	494	494	464	464
In intensive care ward	949	949	949	949	932	932
In resuscitation unit	803	803	803	803	789	789
**Extra consumable costs resulting from complication (€/patient)**	124	124	124	124	125	125
**Conversion costs (€/patient)** †	3840	3840	3840	3840	3840	3840
**Duration of surgery (min)** *	226·0	224	233·0	225·0	180·0	172
**Costs for operating room (€/min)**	6·90	6·90	7·40	7·40	8·40	8·40
**Conversion rate (%)**	8	0	5	0	3	0
**Instrumentation costs (€/patient)**	1626	1626	3365	3365	3244	3244

* Median values.

† Calculation detailed in Methods section. LTME, laparoscopic total mesorectal excision; RTME, robotic total mesorectal excision; ERAS, enhanced recovery after surgery pathway; n.a., not applicable.

### Cost analysis

Implementation of a robotic programme initially led to higher overall costs, with an increase of €2348 per patient in the total cohort from LTME in 2011 (€11 172) to RTME in 2015 (€13 520) (*Table* 4). Introduction of the ERAS programme decreased the costs associated with stays in the surgical ward (mean cost €4199 (95 per cent c.i. 3786 to 4612) for RTME alone *versus* €3428 (3053 to 3804) for RTME with ERAS; *P* < 0·001) and the ICU (mean cost €870 (456 to 1284) *versus* €538 (290 to 786) respectively; *P* = 0·069). Operating room costs were also reduced with the ERAS programme, but not significantly so (median cost €1724 for RTME alone *versus* €1512 for RTME with ERAS; *P* = 0·129). Costs for RTME plus ERAS were €388 higher per patient than for LTME alone (€11 560 and €11 172 respectively), but €1960 lower than for RTME alone (€11 560 and €13 520) (*Tables* 3 and 4).

**Table 4:** Total costs associated with proctectomy for the whole population

	LTME alone (2011) (*n* = 71)	RTME alone (2015) (*n* = 58)	RTME with ERAS (2018) (*n* = 91)	*P* ¶
Cost of stay in surgical ward (€/patient)*	4952	4199	3428	< 0·001#
Complication costs (€/patient)†	1957	3285	2761	n.c.
Cost of ICU stay (€/patient)*	713	870	538	< 0·001**
Instrumentation costs (€/patient)	1626	3365	3244	n.c.
Operating room costs (€/patient)‡	1559	1724	1512	0·281††
Conversion costs (€/patient)*, §	365	77	77	n.c.
Total (€)	11 172	13 520	11 560	n.c.

* Mean values.

† Calculation detailed in Methods section.

‡ Estimated as the median duration of surgery (*Table* 2) multiplied by the cost per min in the operating room (*Table* 3).

§ Approximated from a large series. LTME, laparoscopic total mesorectal excision; RTME, robotic total mesorectal excision; ERAS, enhanced recovery after surgery pathway; n.c. not calculable (individual data not available).

¶ Kruskal–Wallis test.

# *P* = 0·008, LTME *versus* RTME; *P* < 0·001, LTME *versus* RTME + ERAS; *P* < 0·001, RTME *versus* RTME + ERAS.

** *P* < 0·001, LTME *versus* RTME; *P* < 0·001, LTME *versus* RTME + ERAS; *P* = 0·070, RTME *versus* RTME + ERAS.

†† *P* = 0·355, LTME *versus* RTME; *P* = 0·438, LTME *versus* RTME + ERAS; *P* = 0·129, RTME *versus* RTME + ERAS (Mann–Whitney two‐sample test for pairwise comparisons).

When level 1 patients (absence of co‐morbidity) were considered specifically, costs were lower in this group compared with those in the total cohort (*Tables* 4 and 5). Costs were further reduced after the introduction of ERAS: management of RTME via ERAS resulted in 7·2 per cent lower costs than classical laparoscopic surgery (€7716 *versus* €8312 respectively, a difference of €596) (*Table* 5). As for the total cohort, for the level 1 subgroup there was a significant decrease in costs associated with surgical ward stay for RTME plus ERAS compared with RTME alone: mean €2988 (95 per cent c.i. 2642 to 3334) *versus* €4192 (3754 to 4630) respectively (*P* < 0·001).

**Table 5:** Total costs associated with proctectomy in patients with no co‐morbidity (level 1)

	LTME alone (2011) (*n* = 26)	RTME alone (2015) (*n* = 35)	RTME with ERAS (2018) (*n* = 25)	*P* ¶
Cost of stay in surgical ward (€/patient)*	4636	4192	2988	< 0·001#
Complication costs (€/patient)†	0	0	0	n.c.
Cost of ICU stay (€/patient)*	504	298	39	< 0·001**
Instrumentation costs (€/patient)	1626	3365	3244	n.c.
Operating room costs (€/patient)‡	1546	1665	1445	0·140††
Conversion costs (€/patient)*, §	0	0	0	n.c.
Total (€)	8312	9520	7716	n.c.

* Mean values.

† Calculation detailed in Methods section.

‡ Estimated as the median duration of surgery (*Table* 2) multiplied by the cost per min in the operating room (*Table* 3).

§ Approximated from a large series. LTME, laparoscopic total mesorectal excision; RTME, robotic total mesorectal excision; ERAS, enhanced recovery after surgery pathway; n.c. not calculable (individual data not available).

¶ Kruskal–Wallis test.

# *P* = 0·233, LTME *versus* RTME; *P* < 0·001, LTME *versus* RTME + ERAS; *P* < 0·001, RTME *versus* RTME + ERAS.

** *P* < 0·001, LTME *versus* RTME; *P* < 0·001, LTME *versus* RTME + ERAS; *P* = 0·029, RTME *versus* RTME + ERAS.

†† *P* = 0·157, LTME *versus* RTME; *P* = 0·436, LTME *versus* RTME + ERAS; *P* = 0·074, RTME *versus* RTME + ERAS (Mann–Whitney two‐sample test for pairwise comparisons).

**Figure 1:**
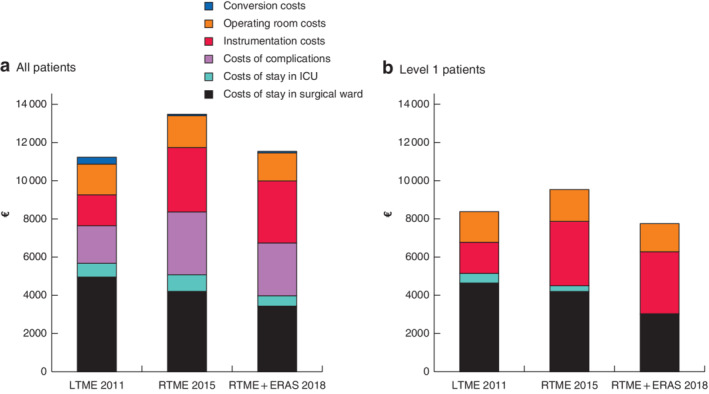
Cost synthesis by items of expenditure LTME, laparoscopic total mesorectal excision; RTME, robotic total mesorectal excision; ERAS, enhanced recovery after surgery.

Cost synthesis by items of expenditure demonstrated that it was not possible to reduce instrumentation costs, whereas the surgeon could have an impact on operating room costs and the cost of stays in the surgical ward (*Fig*. 1).

## Discussion

The introduction of robotic surgery was associated with an initial increase in costs compared with laparoscopy. As experience with the robotic system improves, LOS should decrease and fewer complications will be experienced^13^, ^14^; this will reduce these costs over time. In this study, the learning curve for robotic surgical techniques and implementation of ERAS management were intentionally avoided. Nevertheless, cost‐analysis results indicated that robotic surgery was still more costly than laparoscopy. Although the difference may not be great, cost is a factor that must be considered and can remain a limitation for robotic surgery. Only the introduction of the ERAS programme – after the robotic learning curve had been achieved and the team was familiar with the ERAS procedures involved – enabled costs to be reduced to a level that was comparable to laparoscopy, with lower costs in patients without co‐morbidity.

ERAS management enabled efficiency to be improved (including a significantly shorter operating time and LOS, with lower overall morbidity), so that the overall economic impact of RTME was neutral. This is an important factor to consider for any centre that currently uses, or is thinking of implementing, robotic surgery. In the authors' team, two full years were necessary to obtain such a high compliance (86 per cent) with ERAS (first year, 40 per cent; second year, 54 per cent). The use of the da Vinci® XI robot for the third cohort (RTME plus ERAS) instead of the da Vinci® Si™ robot may explain the shorter operating time and lower morbidity rates in this group.

The cost differences between LTME and RTME have been evaluated in single‐centre studies, which have the advantage of standardized management. A previous Italian study^14^ determined that mean(s.d.) costs were significantly higher for robotic (€9812(1974)) than for laparoscopic (€9045(1893)) surgery (*P* = 0·02). Conversely, there was no significant difference in the costs of hospital stay (€3617(1403) *versus* €3889(1457) respectively; *P* = 0·38). A single‐institute study from Korea^15^ found that total hospital charges were significantly higher for the robotic group (US$14 647 (€13 590; exchange rate 24 March 2020) *versus* US$9978 (€9260) respectively; *P* = 0·001), whereas the hospital profit was significantly lower (US$689 (€640) *versus* US$1671 (€1550); *P* < 0·001). Another group^16^ confirmed a significant reduction in costs with increasing surgeon experience and a fully robotic approach, especially with the da Vinci® Xi system compared with the da Vinci® Si™ robot.

Results from ROLARR^3^, the biggest multicentre phase III study in this field, confirmed that robotic rectal cancer surgery is more expensive than conventional laparoscopic surgery, even after excluding acquisition and maintenance costs for the systems. Nevertheless, the authors emphasized that wide variation in costs is indicative of different practices between surgeons and sites. In addition, the ROLARR trial is often mentioned with relation to the differences in the surgeon learning curve between the two techniques, which can explain some of the results in the robotic group.

With ERAS, RTME has been associated with a shorter LOS and fewer postoperative complications, but longer duration of surgery compared with LTME^17^. The medicoeconomic impact of ERAS for rectal surgery is difficult to demonstrate^18^. However, recent publications have underlined the cost benefit of enhanced recovery after hepatectomy^19^ or pancreatectomy^20^. For colorectal surgery, a systematic review^21^ underlined the poor quality data currently available, but showed that ERAS was less costly and more effective than proctectomy alone.

In the present series, a decrease in costs after the introduction of ERAS was documented. Accordingly, the costs of RTME in 2018 in the full cohort were comparable, but slightly higher, than those for LTME in 2011; in patients with no co‐morbidity, however, the costs were lower for RTME than for LTME.

It must be noted that this study has some limitations. The PMSI database is not a register or an observational database of patients; thus, there is a risk of comparing patient populations that do not have the same clinical characteristics in different periods of time. Furthermore, the implementation of ERAS for LTME was not considered. Finally, economic specificities may vary between countries. On this basis, further studies of larger populations are needed to confirm the medicoeconomic impact of ERAS management following RTME.
